# Domain Knowledge Plus AI Fluency: The New Nonnegotiable Competency for the Health Workforce in the AI Era

**DOI:** 10.2196/100954

**Published:** 2026-10-30

**Authors:** Seble Frehywot, Yianna Vovides

**Affiliations:** 1 Department of Global Health, Milken Institute School of Public Health, The George Washington University Washington, DC United States; 2 Centers in New Design in Learning and Scholarship Georgetown University Washington, DC United States

**Keywords:** artificial intelligence, AI, AI literacy, domain-AI bilingualism, health workforce development, AI system stack, responsible AI integration, digital health

## Abstract

In the ongoing AI boom—now supercharged by large language models and generative AI tools—the rapid integration of AI into health care and public health has created an urgent need for professionals who combine deep domain expertise (clinical, epidemiological, ethical, and contextual expertise in health and public health) with AI fluency (the ability to understand, critically evaluate, use, and oversee AI systems effectively). Without domain guidance, AI risks producing biased, unsafe, or contextually irrelevant outputs that compromise patient safety and exacerbate disparities. Conversely, domain experts lacking AI fluency may underuse or misuse these technologies, missing opportunities to improve care quality, operational efficiency, and population health. A foundational understanding of the modern AI system stack—comprising energy, accelerators, infrastructure, models, and applications—empowers health professionals to make informed decisions on adoption, evaluation, governance, and ethical use without needing to code or build models themselves. This stack framework highlights critical interdependencies and health care–specific challenges, including regulatory compliance, data privacy, sustainability, and resource constraints, particularly in low- and middle-income settings. This paper argues that cultivating domain-AI bilingualism is essential for safe, effective, and equitable AI integration worldwide. It outlines the 5-layer AI system stack applied to health care, key reasons for hybrid expertise, and a practical 4-step pathway: awakening (recognizing AI’s transformative role), intrepidity (courageously engaging foundational AI concepts), bilingualism (translating between clinical needs and AI capabilities), and visionary leadership (driving organizational and policy readiness). It outlines major barriers, targeted strategies, and recommendations for educators, institutions, and policymakers. By bridging domain knowledge with AI fluency, health professionals can direct—rather than be directed by—AI technologies, including emerging agentic systems, toward more precise, sustainable, and human-centered outcomes while mitigating risks. This hybrid capability represents a nonnegotiable competency for the health workforce for thriving in the post-2026 AI era.

## Introduction

In the current AI boom, the rapid integration of AI into health care and public health systems has created an urgent need for professionals who combine deep *domain knowledge* (clinical, epidemiological, ethical, and contextual expertise in health and public health) with *AI fluency* (the ability to understand, critically evaluate, use, and effectively oversee AI tools). This dual competency has become even more critical in the post–large language model (LLM) generative AI era, where LLMs and accessible generative tools have dramatically accelerated AI adoption across clinical, operational, and population health applications. What once required specialized technical teams can now be prompted or fine-tuned by end users in minutes, compressing the timeline for both innovation and potential harm.

Without domain expertise, even sophisticated AI application tools risk generating biased, unsafe, or contextually irrelevant outputs that can compromise patient safety and public health outcomes. Conversely, health domain experts lacking AI fluency may underuse or misuse these technologies, missing critical opportunities to enhance care quality, operational efficiency, and population health. In the ongoing AI boom—now supercharged by generative AI advancements—health and public health practitioners and students require foundational AI knowledge. This is not to transform them into software engineers or coders but to enable them to understand the core layers and components of the modern AI system stack, from data foundations, accelerators (chips), and model architectures to application, deployment, monitoring, and governance.

The imperative holds global relevance. In high-resource settings, domain-AI–fluent professionals are essential for safely scaling precision medicine, hospital operations, and health system optimization. In low- and middle-income countries (LMICs), where health workforce shortages are acute and contextual challenges (such as resource constraints and localized disease burdens) are pronounced, this combination of competencies is vital for equitable and appropriate AI deployment. It helps ensure that globally diffused AI tools do not exacerbate existing disparities but instead strengthen local health systems, support evidence-based decision-making, and address region-specific public health priorities. As generative AI tools continue to proliferate worldwide, building this hybrid expertise across diverse health workforces has become a shared global priority for responsible innovation and sustainable health system strengthening.

This paper examines the key reasons why domain knowledge combined with AI fluency is essential for safe, effective, and equitable AI integration in health and public health; outlines the AI system stack applied to health care and the essential steps required to build domain-AI bilingualism; identifies major barriers to its adoption and practical strategies to overcome them; and offers targeted recommendations for educators, institutions, and policymakers to cultivate this critical workforce capability at scale.

## The 5 Components of the AI System Stack Applied to Health Care

The 5 components of the modern AI system stack (Figure 1 [1])—often described as a “five-layer cake” by industry leader NVIDIA’s Jensen Huang [1]—apply to the health sector as follows. Each layer builds on the one below it to enable scalable, reliable AI in health care.

**Figure 1:**
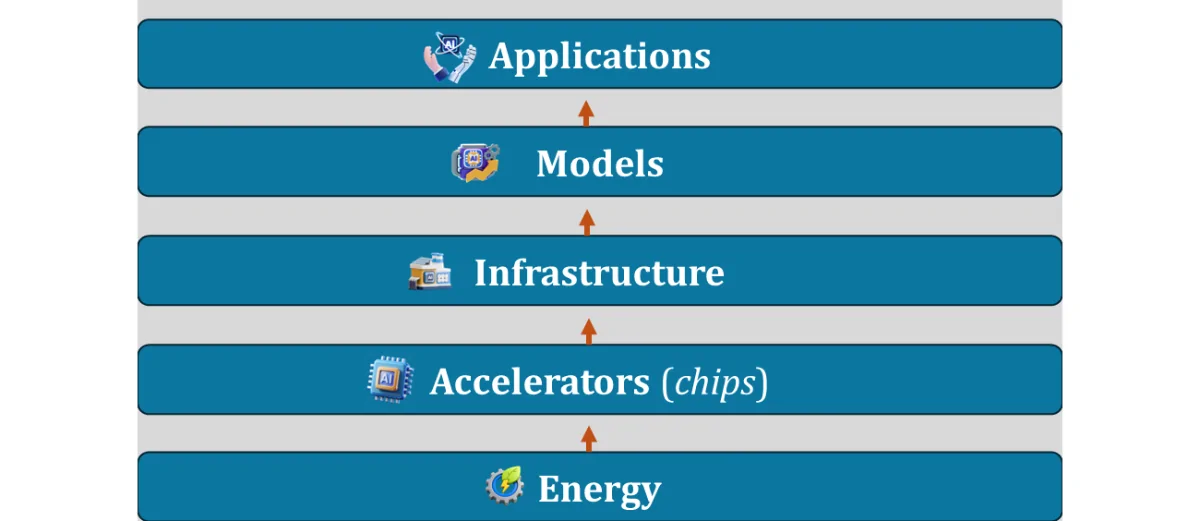
The components of the modern AI system stack in health care (image adapted and created by the authors from Huang [1]).

### Energy

Energy is the foundational power layer. AI systems in health care (especially large-scale training of models or always-on inference in hospitals) consume massive amounts of electricity. Reliable, sustainable energy sources (including efficient grids, renewable energy, and backup power) are critical to avoid outages in life-critical applications such as real-time diagnostics or intensive care unit (ICU) monitoring while also addressing growing concerns about AI’s environmental footprint in medical facilities.

### Accelerators

Accelerators are AI chips. These are specialized hardware such as graphics processing units (GPUs), tensor processing units (TPUs), neural processing units (NPUs), or other AI chips that provide the parallel processing power needed for AI workloads. In health care, these handle compute-intensive tasks such as training models on huge medical datasets or running fast inference for medical image analysis (x-ray, magnetic resonance imaging, computed tomography, and pathology imaging), genomics, real-time edge AI wearables, and portable diagnostic devices.

### Infrastructure

This constitutes the physical and virtual backbone, including data centers, cloud or hybrid setups, high-speed networking, cooling systems, storage, and edge servers. In the health sector, this must comply with strict regulations; support secure handling of sensitive patient data; enable hybrid edge-cloud architectures (eg, on-premises for privacy plus cloud for scale); and power hospital systems, micro–data centers, or AI-ready medical devices.

### Models

These are the AI “brains”—foundation models, domain-specific LLMs, computer vision models, or multimodal models fine-tuned or trained on health care data. Examples include models for radiology report generation, clinical decision support, predictive risk scoring, personalized treatment recommendations, or analysis of electronic health records while preserving privacy.

### Applications

This is the top layer, where AI delivers real-world value to end users. In health care, this includes clinical tools (eg, diagnostic aids and virtual assistants for physicians), operational solutions (eg, predictive bed or staff management and supply chain optimization), patient-facing apps (eg, wearables for monitoring, chat-based triage, and personal care plans), administrative automation (eg, clinical documentation and billing), and population health tools—all designed to improve outcomes, reduce clinician burnout, and enhance access.

### Interdependencies and Health Care–Specific Challenges

The 5 layers of the AI system stack are deeply interdependent in health care, where a weakness in any single layer can cascade into failures across the entire system, directly affecting patient safety, data security, and operational reliability.

Energy and accelerators, for instance, underpin the computational demands of training and inference in large models; unreliable power or insufficient GPU or TPU capacity in hospitals can delay real-time diagnostics or cause outages in ICU monitoring systems. These hardware constraints, in turn, influence infrastructure choices—such as hybrid edge-cloud architectures—where sensitive patient data must be processed locally for speed and privacy while being scaled in the cloud for advanced analytics. Models and applications then rely on this foundation: a multimodal LLM fine-tuned on electronic health records can only deliver trustworthy clinical decision support if the underlying infrastructure ensures secure, compliant data flows and the energy supply remains sustainable.

In resource-limited settings, this interdependence often forces trade-offs between performance (eg, high-accuracy image analysis) and feasibility (eg, edge deployment on portable devices to reduce latency and costs) [2]. Health care introduces unique regulatory and privacy challenges that amplify these interdependencies. Data privacy regulations such as HIPAA (Health Insurance Portability and Accountability Act) in the United States and the General Data Protection Regulation (GDPR) in the European Union (EU) impose strict requirements on patient information across all layers, particularly in infrastructure and models, where vast datasets are ingested for training [3]. Breaches or noncompliant processing—such as using deidentified protected health information in cloud storage or model fine-tuning—can lead to severe penalties and loss of trust [4]. The EU AI Act, which entered into force in 2024, further classifies many health care AI systems (eg, diagnostic tools or predictive risk scorers) as high risk, mandating rigorous conformity assessments, transparency, human oversight, and risk management throughout the stack—from energy-efficient training practices to bias audits in applications [5]. Compliance thus requires domain experts to collaborate closely with technical teams on issues such as secure data governance, explainability of model outputs, and sustainability reporting while navigating extraterritorial implications for global health systems. Without integrated oversight, these challenges risk widening inequities, increasing environmental footprints, or eroding clinician confidence in AI tools. Domain-AI–fluent professionals are therefore essential to navigate this complex landscape responsibly.

## Key Reasons Why Domain Knowledge and AI Fluency Are Essential

This foundational understanding of the components of the AI system stack empowers health care professionals to make informed decisions about adoption, evaluation, governance, and ethical use without needing to build models themselves. It assists them in consciously thinking and acting upon the following aspects.

### Informed Oversight and Critical Appraisal

AI’s impact on the “outcomes of health care” and its “potential to address patient needs” are growing, and this brings ethical and operational challenges [6]. AI models in health care (eg, diagnostic algorithms and predictive tools) can produce errors, hallucinations, or biases if trained on flawed data or misapplied. Domain experts are required to assess accuracy, reliability, validity, and clinical relevance—skills emphasized over pure programming knowledge [7]. Understanding model layers (eg, how transformers process sequences via attention mechanisms) and infrastructure (eg, why GPUs or TPUs are needed for scale) helps identify when outputs are unreliable or when deployment fails due to compute constraints. Without this, there can be overreliance risks, errors, deskilling, or disparities.

### Resource and Sustainability Awareness

Training frontier models requires enormous amounts of energy (often equivalent to thousands of households) and specialized hardware [8]. Practitioners need to grasp these layers to advocate for sustainable AI (eg, prioritizing efficient models or edge inference in resource-limited settings) and assess environmental and public health impacts.

### Effective Collaboration With Technology Teams

Domain experts with foundational AI knowledge can communicate needs, set realistic expectations, and guide validation—asking precise questions about model architecture, training data, accelerators used, or inference costs—leading to better tools and return on investment.

### Ethical, Regulatory, and Risk Management

Knowledge of AI layers helps recognize issues such as hallucinations in LLMs, bias amplification in deep learning, or infrastructure vulnerabilities (eg, data center outages). This supports responsible use, aligning with principles such as human autonomy and transparency. Emerging concepts such as “critical AI health literacy” [9] empower patients and health care professionals to challenge biased systems.

## Building the Bridge: Essential Steps Toward Domain-AI Bilingualism

The journey toward achieving health workforce domain-AI bilingualism requires 4 steps: awakening, intrepidity, bilingualism, and visionary leadership (Figure 2).

**Figure 2:**
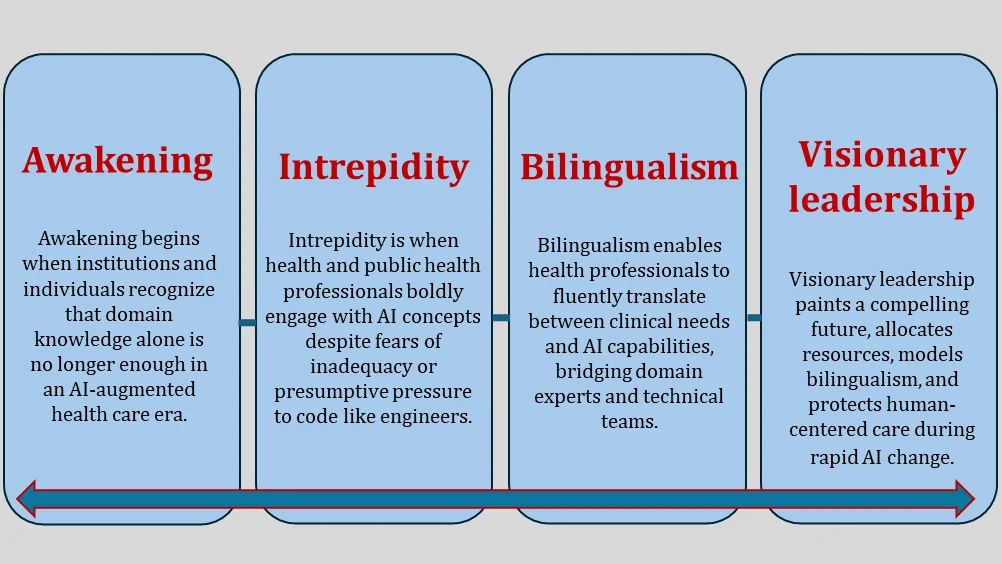
Framework for building the bridge: essential steps toward health workforce domain-AI bilingualism (image created by the authors).

### Awakening to This Reality

Awakening begins with institutional and individual recognition that domain knowledge alone is insufficient in an AI-augmented health care era. Many clinicians and public health leaders still treat AI as a peripheral innovation rather than a transformative force reshaping diagnostics, workflows, and population health strategies. This realization creates urgency and overcomes inertia. For example, a 2025 cross-sectional study of European health care professionals revealed persistent low AI literacy, with over 60% citing lack of understanding of AI capabilities and limitations as a major barrier to adoption and only 13.8% feeling that their training actually prepared them for AI integration; institutions that ran targeted awareness workshops saw rapid increases in voluntary engagement with AI tools [10]. Another case is Singapore’s national health system, which launched a system-wide AI readiness campaign in 2024-2025, including mandatory orientation modules for all clinical staff, which shifted perceptions from skepticism to proactive exploration, resulting in faster pilot approvals for AI-assisted triage systems and measurable improvements in staff confidence [11,12]. Studies show persistent low AI literacy among health care professionals, with barriers such as lack of understanding of AI’s capabilities and limitations, explainability concerns, and fear of professional disruption slowing adoption [13]. Awakening creates urgency, and once there is awareness, motivation emerges for the subsequent steps.

### Intrepidity (Courage to Learn Foundational AI Knowledge Without Fear)

Intrepidity involves clinicians boldly stepping into AI concepts despite fears of inadequacy or expectations to code like engineers. In this context, it means learning about foundational AI concepts. This courage turns early adopters into internal champions who reduce organizational resistance. No one expects clinicians or public health officers to build LLMs, but basic fluency lets them critically assess AI outputs, spot errors in clinical decision support, or use tools ethically [14]. A notable example is the Mayo Clinic’s faculty development initiative, where physicians participated in short, nontechnical “AI literacy bootcamps” focused on understanding model limitations and bias rather than programming [15]. Similarly, nursing programs incorporated hands-on simulation laboratories where nurses experimented with AI chatbots for patient triage without coding requirements [16]. This fearless experimentation approach can lead to frontline staff proposing practical improvements to vendor tools, demonstrating how intrepidity fosters grassroots innovation.

### Bilingualism (Fluency in Both Domain and Foundational AI Language)

Bilingualism equips health professionals to translate seamlessly between clinical needs and AI capabilities, bridging the gap between domain experts and technical teams. This hybrid skill set is the nonnegotiable bridge. Domain experts alone risk misapplying AI (eg, biased models deployed without clinical context); AI experts alone risk producing clinically irrelevant or unsafe tools [17]. This hybrid skill set prevents misapplication of biased models and ensures clinically relevant tools. At Stanford Medicine, interdisciplinary AI–clinical translation teams pair physicians with data scientists in joint rounds; clinicians learn to articulate requirements in terms of model architecture and data needs while engineers gain contextual understanding of ethical and workflow constraints [18,19]. This has produced more accurate radiology AI tools and reduced deployment failures. In the United Kingdom’s National Health Service, a digital health bilingual training pathway for public health officers has enabled professionals to co-design population health dashboards that incorporate epidemiological expertise with multimodal AI models, leading to better-focused interventions during disease outbreaks [20]. Institutions need these translators at every level—clinicians who know the requirements for AI developers and educators who teach foundational AI knowledge–integrated curricula [21].

### Visionary Leadership

Visionary leadership articulates a compelling future, allocates resources, models bilingualism, and safeguards human-centered care amid rapid change. Leaders champion organizational readiness for advanced applications such as agentic AI. They pioneer not only AI fluency for individual readiness but also for organizational readiness [22]. They future-proof the institution—preparing the workforce for agentic AI, personalized care and population health, or AI-driven policy simulation—while preserving humanity in care. A compelling case is Cleveland Clinic’s AI strategy under visionary executives who integrated AI fluency into leadership competencies and established a dedicated Center for Clinical Artificial Intelligence with cross-functional governance [23,24]. Another example is the World Health Organization’s regional initiatives that combine strategic planning with hands-on exposure [25,26]. These efforts help policymakers simulate AI-driven resource allocation scenarios while prioritizing workforce protection, demonstrating scalable visionary impact while preserving humanity in care [27].

These 4 elements—awakening, intrepidity, bilingualism, and visionary leadership—form a progressive pathway that transforms individual mindsets into institutional capability, ensuring that the health workforce thrives alongside AI rather than being displaced by it.

## Potential Barriers and How to Overcome Them

### Overview

Despite the clear imperative for domain knowledge combined with AI fluency, several barriers continue to slow progress in health care and public health settings. Time constraints in already demanding clinical schedules often leave little room for new learning, while persistent fears of professional disruption or “deskilling” foster resistance among practitioners. Many clinicians worry they lack the technical aptitude to engage with AI concepts, and resource disparities—particularly between well-funded academic centers and underresourced community or LMIC settings—risk widening the digital divide. Infrastructure limitations, such as unreliable energy supplies, legacy systems incompatible with modern accelerators, or stringent data privacy requirements under HIPAA or the GDPR, further complicate deployment of even well-designed models and applications. Additionally, fragmented regulatory environments and the rapid pace of AI evolution can overwhelm institutions, leading to cautious or inconsistent adoption [28].

These barriers are surmountable through targeted, layered strategies that align with the 4 foundational elements. Awakening can be advanced via concise, role-relevant awareness campaigns and success stories shared through grand rounds or internal newsletters, gradually shifting mindsets from skepticism to curiosity. Intrepidity is cultivated by designing low-stakes, modular learning experiences—short online modules, peer-led simulation laboratories, and “AI playground” environments—that emphasize critical evaluation over coding expertise, reducing fear of incompetence. Bilingualism grows when institutions create structured collaboration forums, such as joint AI-clinical rounds or translation workshops, enabling domain experts and technologists to codevelop tools and build mutual understanding—in the process building a critical mass of bilingual peer champions. Finally, visionary leadership is key to overcoming systemic hurdles: leaders can secure dedicated funding; advocate for policy support (eg, aligning with the EU AI Act’s high-risk requirements) [29]; and prioritize equitable infrastructure investments, including edge computing solutions for resource-limited environments.

By proactively addressing these challenges with practical, human-centered solutions, health systems can transform potential obstacles into opportunities for sustainable and inclusive AI integration, ensuring that the entire workforce is equipped to harness AI responsibly while preserving the core values of compassionate care.

### Navigating Energy Crises, Workforce Shortages, and Hardware Constraints Through Domain-AI Bilingual Leadership

The case for domain-AI bilingualism is strongest in LMICs facing acute workforce shortages [30] and chronic energy crises—blackouts, unstable grids, and limited renewable energy [31]. South Asian studies document specialist shortages and load shedding that force the adoption of lightweight mobile models [32,33]; Latin American and Global South reviews note interoperability failures, technical brain drain, and nonlocal models that underperform on regional disease patterns [32,33]. Bilingualism must augment scarce capacity rather than add administrative layers, using existing human capital instead of paperwork or overtime [34]. AI fluency should be embedded into routine work through passive, just-in-time learning and shared digital infrastructure so that overstretched workers gain oversight without extra load.

GPUs and TPUs need continuous high-wattage power infrastructure, which is rare in LMIC facilities, and cooling that many district hospitals lack [35,36]; hence, large multimodal models become impractical. Clinics often rely on intermittent grids or diesel, making energy-intensive training impossible and continuous inference unreliable [37]. The result is dependence on foreign models trained on high-resource data, with performance drops and bias against local phenotypes [38]. Edge and TinyML mitigate but do not fully close the indicated gap [39]. Leaders must select lightweight, offline-first architectures on smartphone NPUs or single-board computers drawing under 10 W [40] runnable from small solar panels, car batteries, or weak grids. Options include quantized or distilled models that cut compute by 70% to 85%, on-device inference that avoids continuous connectivity, and hybrids that diagnose locally and sync when power and bandwidth allow [39,41]. The total cost of ownership—diesel vs renewable microgrids—should be assessed, and vendor transparency on energy and hardware should be demanded. Selection then shifts from frontier LLMs to context-appropriate tools that retain utility under instability and stop lower-stack failures from cascading into applications [32]. Cases include offline chest x-ray AI tuberculosis diagnosis in South African mines without cloud or on-site radiologists [42] and India’s offline portable primary health systems where bilingual district officers choose low-power models [43]. Leaders who understand energy and accelerator layers scale tools that remain functional when the national grid fails, so bilingualism strengthens rather than strains scarce human resources.

### Operationalization of Lower-Stack Hardware and Energy Knowledge by Frontline Clinicians, Public Health Officers, and Nontechnical Professionals

Nontechnical professionals operationalize lower-stack knowledge through simple heuristics in existing workflows, not new software or reports. A nurse manager keeps a laminated power status card beside the AI triage tablet: green (stable grid or solar) indicates the full model, amber (intermittent) indicates the lightweight edge mode, and red (blackout) indicates paper protocol. A community health supervisor who knows that accelerators generate heat schedules AI-assisted screening for cooler morning hours to avoid thermal throttling. These practices are transmitted through brief verbal briefings at shift handover.

Frontline clinicians and public health officers apply the same knowledge in low-frequency, high-stakes decisions instead of continuous monitoring. A rural physician evaluating an AI chest x-ray first asks whether the model runs offline on existing low-power devices or needs cloud access that fails in outages [44]. If a clinician uses a portable ultrasound AI package with an NPU that draws 3 to 5 W when the local solar microgrid can sustain only intermittent 50-W loads, the clinician might reject a higher-accuracy, cloud-dependent model for an offline quantized version usable during the daily brownout [45]. During procurement, a hospital medical director compares total energy costs and backup needs and rejects platforms that would overload limited solar capacity or increase diesel dependency, protecting life-critical systems such as ICU monitoring. A district public health officer evaluating an AI outbreak dashboard recognizes that continuous cloud inference would require diesel backup costing more than the intervention’s benefit and, therefore, mandates an offline-first architecture that updates only when connectivity and power coincide. A similar logic is visible in India, where AI-augmented platforms for tuberculosis and infant health have been scaled by embedding tools in existing workflows and keeping frontline officers in command of when inference runs rather than assuming continuous cloud availability [46].

Awakening is typically triggered by one observed failure linked to heat or power—an AI tool unusable during load shedding. After repeated outages, a district nursing supervisor insists that AI triage chatbots include offline and low-power modes and trains staff to fall back to paper protocols when power drops. Intrepidity appears when staff test the heuristic under real conditions or clinicians request vendor demonstrations under simulated brownout. Awareness of accelerator and energy demands then favors quantized edge models over high-accuracy but power-hungry alternatives, preserving availability during night shifts or generator shortages [47]. Bilingualism develops when the nurse manager states the heuristic to procurement in wattage and thermal limits or a primary care physician reviewing vendor proposals asks about inference hardware and energy per encounter and rejects cloud-only solutions that would exclude satellite clinics, yielding language such as “must sustain 4-hour offline inference on a 10 W NPU.” Visionary facility leadership formalizes the heuristic as a standard operating procedure and embeds hardware-aware criteria in technology assessment checklists so that every future purchase is energy aware without new administrative burden. These actions—procurement criteria, training content, and contingency planning—translate stack awareness into practical safeguards without coding expertise [48]. Reviews of trustworthy AI adoption in Indian health care argue that frontline criteria—energy reliability, offline fallback, and workflow fit—are as decisive as model accuracy if safety and ethical use are to be sustained [49]. Parallel Nigerian examples show that such operationalization sustains AI utility under chronic infrastructure stress [50].

## Agentic AI: Deskilling, Automation Bias, and Governance of Agentic Workflows

### Overview

As agentic systems plan and act with less supervision, users trust usually correct outputs—automation bias that turns reviewers into rubber stamps. The handoff deskills oversight: practice migrates to the machine, judgment atrophies, and the human “in the loop” cannot reliably catch errors or override high-stakes steps. Governance cannot treat a token checkpoint as enough; it must keep evaluative agency alive through scoped permissions, irreversible action gates, and training that preserves foundational skills so that accountability is real, not theatrical.

### Critical Caveat on Deskilling and Automation Bias With Agentic AI

Visionary claims for agentic AI must be tempered by deskilling and automation bias risks. Multistep autonomous planning [51] leads to progressive deskilling and bias, and repeated AI assistance erodes independent skills [52]. Clinicians who defer to sequential agent recommendations lose the pattern recognition and uncertainty tolerance that constitute clinical intuition [53]. For trainees, the risk is “never-skilling”: formative diagnostic practice is displaced, and cognitive offloading further weakens those skills [52,54]. Psychologically, high-confidence agent outputs produce complacency, reduced vigilance, and eroded self-efficacy—already observed with earlier decision support and amplified by agentic continuity across a care episode [54]. Cognitively, the human-in-the-loop role shifts from active reasoning to passive verification, accelerating decay especially among early-career practitioners [55]. Awareness of this produces mitigation intrepidity, which requires mandatory independent reasoning before viewing agent recommendations, periodic “AI-off” practice, and curricula that treat agents as copilots whose failures bilingual clinicians must detect and override. Without safeguards, agentic advances risk developing a workforce less able to intervene when systems fail or hit edge cases.

### Governance, Ethical, and Clinical Validation Protocols Specific to Agentic Workflows

Agentic systems that chain reasoning and action need governance unlike that for passive diagnostic tools [56]. Validation moves from single-output accuracy to end-to-end process evaluation under realistic resource constraints: final accuracy, intermediate decision quality, recovery from partial failures, and energy and accelerator use across the trajectory [51]. The human-in-the-loop role must be substantive (named override authority and predefined escalation thresholds), not nominal [55].

Multiagent interactions create compounded opacity and diffuse accountability; protocols must mandate full-chain explainability, bias and equity audits throughout, transparent disclosure to preserve patient autonomy, and explicit liability [56]. Ethical rules require continuous human override, audit logging of every step, and bias audits of whether multistep plans disadvantage underrepresented populations or low-resource facilities [57]. Governance needs a control plane: agent identity and persona registries, least-privilege tool access, continuous logging of actions and intermediate reasoning, human authorization gates for consequential acts, and life cycle management with decommissioning tied to credential revocation [58]. Frameworks should mandate agentic risk committees that include domain-AI bilingual clinicians and a health workforce empowered to pause or redesign workflows when energy, privacy, or equity thresholds are breached [59]. These controls keep domain experts in meaningful oversight of systems whose autonomy amplifies benefit and harm [58].

## Recommended Actions for Educators, Institutions, and Policymakers

### Curriculum Integration and Faculty Development Programs

#### Overview

Educators play a pivotal role in embedding AI fluency into health professions training without displacing core clinical and public health curricula. Rather than teaching programming, programs should integrate modular, foundational AI literacy courses that explain the full AI system stack, model limitations (such as hallucinations and bias), and critical appraisal techniques tailored to health care contexts. Medical, nursing, and public health schools can adopt case-based learning where students evaluate real-world AI tools for diagnostic accuracy, ethical risks, and implications. For instance, simulation exercises using deidentified datasets allow learners to practice “bilingual” translation—articulating clinical needs to hypothetical AI developers—preparing them for collaborative practice. Faculty development is equally essential; institutions should offer accessible workshops similar to those implemented at Mayo Clinic and other centers to help educators themselves achieve AI fluency and redesign curricula progressively [60].

#### Concrete Competency Matrices for Modular AI Literacy Courses

Modular foundational AI literacy needs concrete competency matrices to fit dense curricula. Medical and public health programs require explicit minimum thresholds by training stage [61]. Drawing on expert consensus and frameworks [61-63], a progressive structure follows.

Training for third-year medical students (preclinical and early clinical stage) should focus on foundational awareness: knowledge (explain the 5-layer AI system stack conceptually, recognize common failures [hallucination, bias, and data shift], and describe basic evaluation [accuracy, fairness, and explainability]), skills (appraise a published AI report or demonstration using a simple checklist and communicate AI use and limits to patients in simulation), and attitudes (maintain human oversight and ethical awareness). Assessment should be via short standardized tests and situational judgment items. No coding or hardware procurement is required.

Training for specialist residents should focus on all prior competencies plus deeper application: integrating AI outputs while documenting independent reasoning, evaluating hardware and energy feasibility for specialty tools, joining local validation or bias audits, and teaching juniors. Technical use includes operating point-of-care devices and recognizing when infrastructure invalidates results. Assessment should be via objective structured clinical examination stations with AI scenarios, critical appraisal portfolios, observed clinical integration, and simulated governance review of an agentic workflow flagging automation bias risks [64].

Awakening embeds the student matrix in existing systems-based courses rather than new modules. Intrepidity uses low-stakes simulation laboratories with offline models. Bilingualism grows when residents colead AI translation rounds with data scientists. Visionary school leadership maps competencies to accreditation standards and gives faculty protected time to reach the resident-level matrix first. Pilots in multiple countries show that graduated thresholds fit dense curricula as modular blocks across existing rotations [65-67].

### Organizational Strategies

Institutions must move beyond pilot projects to systematic organizational readiness. Hospitals and public health agencies should establish interdisciplinary AI governance committees that include domain experts at every decision level, ensuring that the AI system stack is evaluated holistically—from sustainable energy sourcing and compliant infrastructure to bias-mitigated models and user-centered applications. Practical steps include conducting AI maturity assessments, investing in secure hybrid infrastructures that respect region or country AI laws, and creating internal “AI champion” networks drawn from intrepid early adopters. Leadership should also prioritize metrics for success, such as reduced clinician burnout through effective administrative AI and improved population health outcomes. Visionary institutions such as Cleveland Clinic have demonstrated that dedicated AI centers combined with clear ethical frameworks accelerate responsible adoption while protecting core human elements of care [68].

### Policy Recommendations

Policymakers and accrediting bodies need to formalize AI fluency as a core competency. This includes updating medical and public health education accreditation standards to require foundational AI knowledge; supporting national AI health literacy frameworks; and incentivizing sustainable, equitable AI deployment through funding and regulatory guidance. The EU AI Act provides a strong model by classifying health care applications as high risk and mandating transparency, oversight, and risk management; similar approaches adapted locally can prevent fragmented adoption [69,70]. Policymakers should also address global disparities by funding open educational resources, cross-border collaboration platforms, and infrastructure grants for low-resource settings. By aligning incentives—such as tying reimbursement or certification to demonstrated AI governance—policymakers can accelerate the cultural shift toward domain knowledge combined with AI fluency as the new nonnegotiable standard, ultimately ensuring that AI amplifies rather than undermines safe, equitable, and human-centered health care.

## Conclusions

The AI boom will amplify health care and public health impact only when domain experts actively drive the use of these technologies—ensuring safety, clinical relevance, and human-centered outcomes—rather than allowing technology to lead unchecked.

The modern AI system stack, from energy foundations through accelerators, infrastructure, models, and applications, provides a practical framework for health professionals to understand, evaluate, and oversee AI tools without needing to become coders or engineers. By cultivating awakening, intrepidity, bilingualism, and visionary leadership, students, practitioners, educators, and institutions can bridge the current literacy gaps and build the nonnegotiable hybrid competency required for the AI era. Educators must integrate foundational AI concepts into curricula, institutions should establish robust governance and collaborative structures, and policymakers need to formalize fluency requirements and equitable access frameworks. Addressing barriers such as time constraints, fear, resource disparities, and regulatory complexity through targeted strategies will accelerate this transformation and prevent fragmented or inequitable adoption.

Looking forward, the rise of agentic AI—autonomous systems capable of planning, reasoning, and executing multistep tasks—promises profound advancements in personalized medicine. Agentic AI could integrate multimodal patient data, real-time sensor inputs from wearables, and population-level insights to generate dynamic, adaptive care plans tailored to an individual’s genetics, lifestyle, environment, and evolving health status. In public health, these systems could simulate policy scenarios at scale, optimizing resource allocation during outbreaks or chronic disease prevention campaigns. However, realizing this vision demands vigilant attention to disparity: without deliberate design, agentic AI risks exacerbating disparities if trained predominantly on data from high-resource settings, deployed unevenly across regions, or governed without diverse domain expertise. Energy sustainability, robust privacy protections across the full AI stack, and inclusive governance will determine whether these technologies narrow or widen global health gaps. Visionary leaders and bilingual professionals must therefore champion ethical guardrails, continuous oversight, and human-in-the-loop frameworks that preserve compassion, autonomy, and trust at the heart of care. Ultimately, the future of health and public health lies not in replacing human expertise with AI but in empowering domain-fluent professionals to direct AI tools toward more precise and humane outcomes for all.

## Abbreviations

EU: European Union
GDPR: General Data Protection Regulation
GPU: graphics processing unit
HIPAA: Health Insurance Portability and Accountability Act
ICU: intensive care unit
LLM: large language model
LMIC: low- or middle-income country
NPU: neural processing unit
TPU: tensor processing unit
